# Thoracic trauma in the geriatric population and possible preventive measures: a retrospective analysis of 261 cases

**DOI:** 10.55730/1300-0144.5880

**Published:** 2024-06-01

**Authors:** Mehmet ÇETİN, Necati SOLAK, Büşra ÖZDEMİR ÇİFLİK, İlteriş TÜRK, Sebahattin Sefa ERMANCIK, Koray AYDOĞDU

**Affiliations:** 1 Department of Thoracic Surgery, Ankara Etlik City Hospital, Ankara, Turkiye; 2 Department of Thoracic Surgery, Mardin Training and Research Hospital, Mardin, Turkiye; 3 Department of Thoracic Surgery, Ataturk Sanatoryum Training and Research Hospital, University of Health Science, Ankara, Turkiye

**Keywords:** Geriatrics, Itaki Fall Risk Scale, rib fracture, thoracic trauma

## Abstract

**Background/aim:**

The increase in the proportion of the elderly population within society is concurrently escalating their vulnerability to traumas, notably falls associated with age-related comorbidities.

**Materials and methods:**

This retrospective analysis involved the examination of data pertaining to patients aged 65 and above who were admitted to our clinic for inpatient treatment following thoracic trauma. Various parameters were statistically compared between the groups with indoor and outdoor traumas.

**Results:**

Of the 261 patients included in the study, 59.4% were male, and the average age in the entire sample was 75.52 ± 7.79. Moreover, 136 (52%) patients had indoor trauma, while 125 (48%) had outdoor trauma. The mean value for all the patients on the Itaki Fall Risk Scale (FRS) II score was 11.04 ± 4.18. The Itaki FRS II score was significantly higher for indoor accidents (11.90 ± 4.34) compared to outdoor accidents (10.10 ± 3.78) (p < 0.001). Additionally, the absence of a fall history and low risk according to the Itaki FRS II score were higher for outdoor accidents compared to indoor accidents, and the difference was statistically significant (p < 0.001). In geriatric trauma occurring outdoors, bilateral rib fractures and extrathoracic findings were significantly more prevalent (p = 0.011 and p = 0.010, respectively). The majority of patients were followed-up without any surgical intervention (73.9%), the most common surgical interventions were catheter (10.3%) and tube thoracostomy (10.3%), and 1.5% of the patients required surgical exploration. Trauma resulted in mortality in 1.5% of the patients.

**Conclusion:**

In the future, specialized measures and prospective studies tailored to the geriatric population, which will constitute the largest demographic segment of society, can facilitate the prevention of trauma-related morbidity and mortality, including associated financial costs.

## 1. Introduction

As the average life expectancy increases due to improved living standards, medical care opportunities, and advancing technology, there is a significant increase in the geriatric population. Additionally, the declining birth rate in developed and developing countries has led to an increased density of the geriatric age group in these regions. It is projected that by 2030, the geriatric age group in western countries will constitute 25% of the total population, and by 2035, the geriatric population will surpass the pediatric age group [1].

This increase in the geriatric population brings several age-related challenges. The burden on health and insurance systems, particularly due to chronic diseases, increases with the elderly population. Concurrently, hospital admissions are increasing, primarily due to indoor and mechanical falls. When evaluated alongside the chronic diseases prevalent in the geriatric population, these falls can lead to significant morbidity and mortality. Indoor falls contribute to increased morbidity, particularly due to prolonged bedridden periods, limited mobility, the need for care, and additional pathologies triggered by these conditions, such as depression [2].

It was reported that 28% of trauma-related deaths occur in the geriatric population, with trauma predominantly related to traffic accidents and falls. Studies in the literature have examined morbidity and mortality and evaluated the effectiveness of trauma scores for both general trauma and isolated thoracic trauma in individuals over the age of 65. Furthermore, protective measures against falls, which are associated with weakened coordination and reflexes in this age group, have been extensively discussed. Simply being over the age of 65 is considered a minor risk factor for falling [3]. Although indoor falls are predominantly low-energy traumas, they can result in increased morbidity and mortality in the geriatric population, similar to outdoor falls [4]. Consequently, the literature includes a wide range of preventive recommendations for falls in the elderly population, such as regular screenings, balance training, vitamin D supplementation, routine eye examinations, and home modifications [5].

The current study aimed to assess the proportion and impact of falls among the elderly population that could be mitigated through preventive measures, with a particular focus on indoor falls.

## 2. Materials and methods

Before beginning the study, approval was obtained from the Ankara Etlik City Hospital local ethics committee (approval number AEŞH-BADEK-2024-197). The study was planned and conducted in accordance with the Declaration of Helsinki.

The patients’ data were retrospectively extracted from the hospital information system. All trauma patients aged 65 years and older admitted to the thoracic surgery clinic due to thoracic trauma were included in the study. Patients over 65 years of age without a history of trauma, those not intended for hospitalization following trauma, and those referred to our hospital after the earthquake in Kahramanmaraş on February 6th, 2023, were excluded. In addition, patients for whom data could not be accessed or were incomplete through the hospital system were also excluded. Demographic characteristics, anamnesis, routine blood results, radiological images, hospitalization notes and, if operated on; surgical reports of patients over 65 years of age with geriatric thoracic trauma who were treated as inpatients in our thoracic surgery clinic between September 1st, 2022, and February 1st, 2024, were retrospectively scanned.

In thoracic trauma cases, all patients with more than one rib fracture in the geriatric population were initially monitored in the thoracic surgery intensive care unit.

The age, sex, and history of chronic diseases of the patients were examined along with other factors such as the cause of trauma, the affected side, anticoagulant use, leukocyte count, hemoglobin level, coagulation time, creatine kinase level, serum potassium values, areas of rib and sternum fractures, additional extrathoracic pathologies due to trauma, thoracic and extrathoracic complications, surgical treatment (if applicable), length of hospital stay, duration of drainage, intubation status, mortality, Itaki Fall Risk Scale II (FRS II) score, and Glasgow Coma Scale (GCS) score.

The Itaki FRS II is a mandatory form to be filled out for all inpatients aged 18 and over admitted to the hospital, as it is among the Quality Standards published by the Ministry of Health of the Republic of Türkiye. When determining the risk level, parameters such as age, level of consciousness, fall history, diseases/comorbidities (hypotension, vertigo, cerebrovascular disease, Parkinson’s disease, limb loss, seizures, arthritis, osteoporosis, fractures), mobility, elimination needs, visual status, medication use, and equipment use (any equipment restricting movement, e.g., intravenous infusion, urinary catheter, chest tube, etc.) were utilized. Scores between 0 and 9 are considered low risk, while scores of 10 and above are considered high. For high-risk patients, a sign stating High Fall Risk Patient was placed at the entrance of their room [6]. Since all the parameters were present in the retrospectively analyzed data of the patients, the data were processed by excluding that related to altered consciousness due to trauma at the time of admission, if present, along with the use of equipment applied in the hospital, to calculate the Itaki FRS II scores.

The GCS was used to evaluate the brain functions in patients admitted to the hospital. It consists of three categories: eye-opening, verbal response, and motor response parameters. The scoring ranges from one to four for eye-opening, one to five for verbal response, and one to six for motor response. The minimum GCS score that can be obtained is 3 and the maximum is 15. [7].

The findings obtained from this study were grouped as indoor and outdoor trauma according to the causes of trauma in geriatric patients who were exposed to thoracic trauma. Falling from the same level, falling in the bathroom, falling from a bed and chair, and trauma after lifting were included in the indoor trauma group. In and out of vehicle traffic accidents, falling from a height and down the stairs, assault, animal kick, and trauma after CPR were evaluated in the outdoor trauma group.

All the analyses were conducted using IBM SPSS Statistics for Windows 24.0 (IBM Corp., Armonk, NY, USA). Descriptive statistics were expressed as the number (n), percentage (%), and mean ± standard deviation (SD) for age. Normality tests were conducted for the duration of hospitalization, duration of drainage, number of rib fractures, and Itaki FRS II score. For the normally distributed variables, such as the number of rib fractures and the Itaki FRS II score, the results were expressed as the mean ± SD. For the nonnormally distributed variables, such as the duration of hospitalization and drainage, the results were expressed as the median (min–max). For the comparison of two groups based on the type of trauma (indoor and outdoor trauma), an independent two-sample t-test was used for normally distributed continuous numerical variables, and the Mann–Whitney U test was used for nonnormally distributed continuous numerical variables. Additionally, Pearson’s chi-squared analysis was used to compare the distributions of the categorical variable groups based on the cause of trauma (indoor and outdoor trauma). p < 0.05 was considered statistically significant.

## 3. Results

There were a total of 277 geriatric trauma patients. Of these, 16 were excluded from the study because they were earthquake victims. Of the remaining 261 patients, 155 (59.4%) were male and 106 (40.6%) were female. The average age of the patients was 75.52 ± 7.79 years and the number of patients aged 85 and over was 40 (15.3%).

In terms of the number of patients with chronic diseases, 62 (23.8%) had no chronic disease, 65 (24.9%) had only one, 64 (24.5%) had two, and 41 (15.7%) had three. In 29 (11.1%) patients, there were four or more chronic diseases. The most common chronic diseases were, respectively, hypertension in 134 patients, diabetes mellitus in 78 patients, coronary artery disease in 44 patients, hyperlipidemia in 18 patients, chronic obstructive pulmonary disease in 16 patients, cerebrovascular disease in 15 patients, congestive heart failure in 12 patients, and chronic kidney disease in eight patients. Moreover, 83 (31.8%) of 261 patients were using anticoagulants. The causes of trauma were grouped into indoor and outdoor accidents and are presented in Table 1.

The mean value for all the patients on the Itaki FRS II was 11.04 ± 4.18. When categorized according to the Itaki FRS II scores into low and high risk, 90 (34.5%) patients were low risk, while 171 (65.5%) patients were high. When the fall histories of the last six months were evaluated, 170 (65.1%) patients had a history of falling, whereas 91 (34.9%) did not. The comparison of the number of falls in the last six months and the results of the Itaki FRS II between the indoor and outdoor geriatric traumas are shown in Table 2.

Considering the average blood values ​at the time of hospital admission, that for hemoglobin was 12.60 ± 1.78 g/dL, for the white blood cell count was 10.12 ± 3.72 × 103/μL, for international normalized ratio was 1.12 ± 0.35, for serum potassium was 4.39 ± 0.55 mmol/L, and the mean creatinine kinase level was 364.48 ± 487.09 U/L.

**Figure 1:**
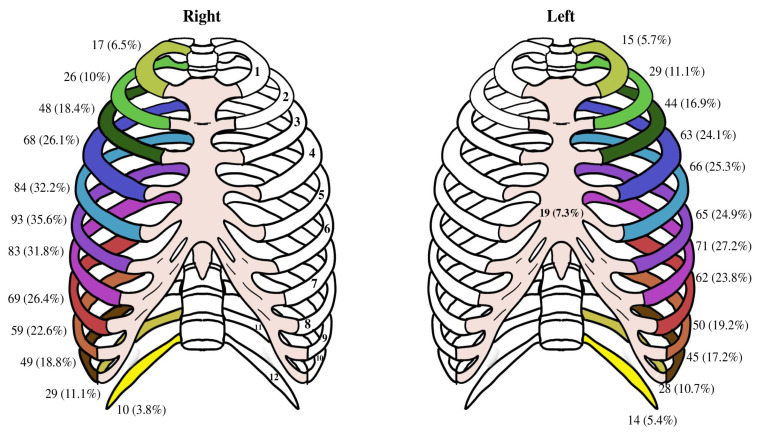
Fractured ribs after trauma in the geriatric population.

**Figure 2:**
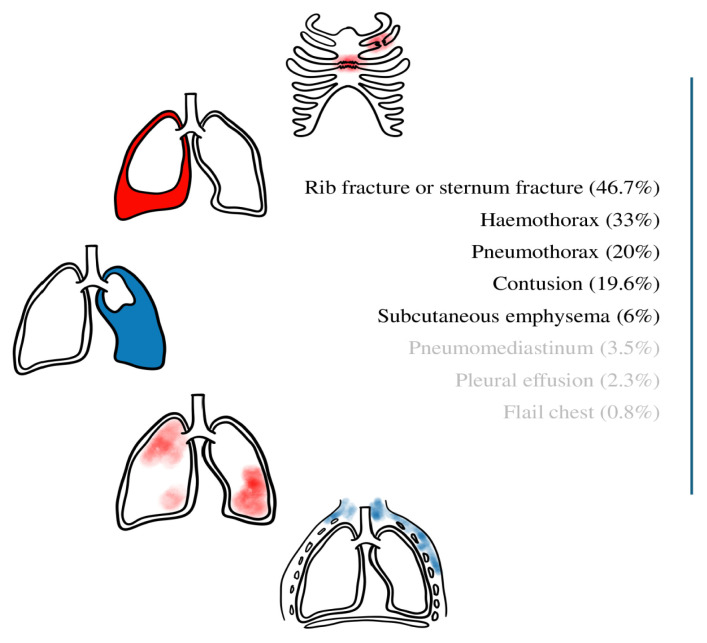
Thoracic trauma presentations in the geriatric population.

The average number of rib fractures was 4.57 ± 2.83. The relationship between the patient count and rib and sternum fractures is shown in Table 3. In addition, the number and location of the broken ribs and total rate in all the patients are shown in Figure 1. Additionally, the proportion of all thoracic findings following trauma is shown in Figure 2.

While no intrathoracic findings in addition to rib or sternum fracture were observed in 122 (46.7%) patients, hemothorax was detected in 86 (33%) patients, pneumothorax in 52 (20%), contusion in 51 (19.6%), subcutaneous emphysema in 17 (6%), pneumomediastinum in nine (3.5%), pleural effusion in six (2.3%), and flail chest in two (0.8%).

While 193 (73.9%) patients were followed-up without any surgical intervention, 27 (10.3%) received catheter thoracostomy, 27 (10.3%) received tube thoracostomy, eight (3.1%) received intercostal blockage, and five (1.9%) received thoracentesis. Moreover, fiberoptic bronchoscopy was performed in three (1.2%) patients and four (1.6%) were operated on.

According to the data obtained, 169 (64.8%) patients experienced isolated thoracic trauma, while extra thoracic trauma was detected in 92 (35.2%) patients. Among those with extra thoracic injuries, 47 (18%) required evaluation by neurosurgery, 19 (7.3%) by orthopedics, and two (0.8%) each by general surgery, otorhinolaryngology, and plastic surgery. Additionally, one (0.4%) patient required evaluation by ophthalmology. Furthermore, in 19 (7.1%) patients, assessment by a nonthoracic trauma department was required for more than one specialty.

Of the 261 patients with geriatric thoracic trauma, four (1.5%) required intubation and four (1.5%) died. Among the intubated patients, two (0.8%) experienced cardiac arrest, while the remaining two (0.8%) had desaturation. The average length of hospital stay for all the patients was 4.63 ± 2.56 days. Among the 55 patients who received a drain, the average duration of drain retention was 4.35 ± 1.72 days. Traumatic incidents were categorized into indoor and outdoor traumas, and the data obtained from the comparison of variables are presented in Table 4.

## 4. Discussion

Syncope of cardiovascular and neurological origin, decrease in visual acuity and reflexes, and increased balance disorders due to loss of muscle strength play significant roles in trauma-related hospital admissions among the elderly population. Age-related bone stiffness and osteoporosis elevate the risk of even minor traumas, particularly rib fractures, while anticoagulant use for comorbidities may exacerbate accompanying intrathoracic complications, notably hemothorax [1,8,9]. Sarcopenia, characterized by progressive muscle function loss due to skeletal muscle mass decline, affects 24%–56% of individuals over 60 years of age and contributes to increased falls and related consequences [10]. In the current study, a statistical disparity was observed in the number of patients hospitalized due to indoor, low-energy traumas compared to outdoor, high-energy traumas. This discrepancy underscores the vulnerability of the geriatric population to thoracic traumas regardless of trauma energy level. Although no discrepancy exists in accompanying intrathoracic findings, the higher prevalence of findings in indoor accidents reinforces this observation. Furthermore, while only 62 (23.8%) patients had no chronic diseases, the fact that the number of patients with three or more chronic diseases (n = 80, 26.8%) exceeded this group suggests the heightened risk of falls and associated pathologies in the geriatric population. Moreover, the use of anticoagulants by 83 (31.8%) patients indicates a likely correlation with the high incidence of accompanying hemothorax (n = 88) due to comorbidities. In the current geriatric thoracic trauma cases, a similar number of rib fractures (n = 4) was observed in both the indoor and outdoor cases. This similarity in the rib fracture incidence, despite low-energy traumas indoors, aligns with the current literature, suggesting a more delicate bone structure predisposing individuals to bone tissue damage [11].

While the primary focus of this study was on the impact of low-energy traumas on the elderly population, the prevalence of bilateral rib fractures and associated extrathoracic findings, particularly in high-energy traumas, holds significance for validating data consistency.

In the present study, since there was no patient follow-up after discharge, only the hospitalization periods were evaluated and when mortalities were examined, a mortality rate of 1.5% (n = 4) was observed in the entire patient group. Özdemir and Köse reported a mortality rate of 4.8% in the geriatric population after thoracic trauma. Moreover, Neupane et al. stated that the mortality rate in the geriatric population with multiple rib fractures was two to five times higher than in young people [2,8]. The absence of a young population in the current study did not allow for a comparison to be made. In addition, it is thought that our clinical approach, as suggested by Özdemir and Köse, is that geriatric rib fracture cases are first followed-up in the intensive care unit and then transferred to the ward after they are stable, which increases the quality of patient follow-up and decreases mortality [8]. Furthermore, in a study by Pyke et al. [12], in which they evaluated 258 cases of thoracic trauma, it was determined that the evaluation of trauma cases in the intensive care unit in the geriatric population was more positive in terms of patient outcomes.

In the literature, it has been noted that factors influencing mortality encompass hemothorax accompanying rib fractures, the quantity of broken ribs, and accompanying extrathoracic traumas [11,13]. However, as there were only four mortalities observed in the present study, further comments could not be provided. Among these cases, two patients experienced cardiac arrest, while intubation and subsequent death resulted from respiratory distress in the remaining two patients.

Although sex did not exhibit a direct statistical correlation with falls in the current study, a relatively higher proportion of females was observed to experience indoor falls. This trend may be attributed to socioeconomic factors and cultural norms prevalent in Türkiye. It is well-established that elderly females tend to lead more indoor-centric lifestyles compared to their male counterparts [13].

The fact that trauma, even if it is low energy, can lead to hospitalization and even intensive care admission in the elderly patient group requires a clear explanation of the risk factors, because these kinds of traumas can be seen in a wide range of patient groups, where one or almost all of a multivariate group of risk factors such as age, cognitive status, previous falls, balance problems, medical history, hearing and vision defects, and structural hazards of the house and building might be present together [14,15].

The increasing health needs and additional problems of the geriatric population after a fall appear both as an increased burden on the healthcare system along with the increase in the elderly population, and as a comorbidity factor that can be eliminated with simple measures. Simple screening and monitoring programs and the elimination of slippery floors, inadequate lighting, hard and angular indoor furniture, and unsupported toilets and bathrooms are simple but effective measures that can be taken in the geriatric population. In addition, choosing relatively soft floors instead of hard floors would minimize the damage that might occur, even after a trauma [13].

According to a report by the World Health Organization, approximately 30% of people over the age of 65 fall at least once every year. Although not all of these falls constitute a hospital admission, it is known that approximately 40 thousand patients die annually due to falls in the USA [16].

Different scales have been developed worldwide for identifying fall risk and implementing preventive measures. In Türkiye, the commonly used fall scales are the Morse Fall Scale, Itaki FRS II, and Hendrich II Fall Risk Model. In comparative studies in the literature, the superiority of these scales over each other has been demonstrated in terms of sensitivity and specificity [6,17]. Among these, the Itaki FRS II is routinely used in our hospital. The primary goal of these scales is to guide the identification of fall risk and the implementation of preventive measures [6]. A fundamental limitation of the scoring performed at hospital admission is that the scores in certain areas, such as equipment use, may be elevated due to interventions, especially in post-fall assessments. Nonetheless, we believe these scores are valuable in providing a general overview of the patients. Indeed, upon evaluating the study data, it was observed that there was a significantly high proportion of both the score and being in the high-risk group among patients with indoor falls. Additionally, the fact that 76% of the indoor patients and 65.2% of all the patients had a fall history in the last six months is particularly significant. This data indicates that significant improvement can be achieved, especially in indoor falls, with preventive measures in place for patients.

In one review, various evaluations and solutions were given by researchers, especially from developed countries. When related studies were researched in the review, it was determined that there have been many detailed evaluations regarding falls, but even they were far from providing personalized recommendations, especially for the geriatric population with impaired cognitive functions. It was also emphasized that there is a lack of reliable data on this subject from developing countries, such as Türkiye, and underdeveloped countries [18].

There have been several innovative studies such as the use of wearable sensors, prefrontal cortex stimulation, near-infrared spectroscopy, exercises (exergames, volitional step training, reactive step training), vitamin D supplements, medical treatments for osteoporosis and loss of muscle strength, and safe ground to prevent or minimize the damage of traumas. Moreover, these innovations, in their broader and improved forms, can help prevent morbidity and mortality that might occur. However, as mentioned by Lord and Close [19], it seems that studies on falls and preventive measures should be carried out more actively in developing and underdeveloped countries.

The main contribution of the current study is that when all the geriatric patients with thoracic trauma were evaluated, rib fractures and hospitalizations were shown to be as common as those in high-energy trauma patients, even when the trauma was of low energy. This finding reveals that almost half of the falls in the geriatric population can be directly prevented with simple measures, highlighting the importance of preventive measures. However, the study also had some limitations. Due to the retrospective nature of the study, data on vitamin D levels, osteoporosis, and medications other than actively used anticoagulants could not be included. This was considered a limitation, especially since the data used in the Itaki FRS II score calculation were obtained retrospectively and could vary due to the patient data being recorded at the time of admission.

## 5. Conclusion

Indoor injuries in the geriatric population can lead to morbidity and mortality rates as high as those resulting from outdoor injuries, contributing to increased hospitalizations and escalating both public health and healthcare system costs. Therefore, preventive measures against indoor falls among the geriatric population are crucial. We believe that prospective studies using fall scales before the occurrence of fall incidents requiring hospitalization will be effective in reducing falls and the subsequent morbidity/mortality, particularly in the geriatric population.

## Figures and Tables

**Table 1:** Causes and classifications of trauma.

	n	%
Indoor traumas	136	52
Fall from the same level	108	41.4
Falling in the bathroom	11	4.2
Falling from bed	8	3.1
After lifting	6	2.3
Falling from a chair	3	1.1
Outdoor traumas	125	48
In-vehicle traffic accident	51	19.5
Falling from a height	32	12.3
Out-of-vehicle traffic accident	16	6.1
Falling down the stairs	16	6.1
Assault	4	0.4
Animal kick	4	1.5
After CPR	2	0.8

**Table 2:** Comparison of Itaki FRS II data for the indoor and outdoor geriatric trauma cases.

Parameter	Geriatric trauma		Statistical analysis

	Indoor	Outdoor	p-value

Itaki FRS II score	11.90 ± 4.34	10.10 ± 3.78	<0.001

Itaki FRS II group			
Low risk	32 (23.5%)	58 (46.4%)	<0.001
High risk	104 (76.5%)	67 (53.6%)	

Fall history in the last 6 months			
No	32 (23.5%)	59 (47.2%)	<0.001
Yes	104 (76.5%)	66 (52.8%)	

**Table 3:** Rib and sternum fracture distribution in the patients.

	Patient count (n)	%
Rib fractures (side)		
Right side	118	45.2
Left side	92	35.2
Bilateral	51	19.5
Rib fractures (number)		
None	10	3.8
Single	13	5
1–5	162	62.1
>5 (max: 13)	76	29.1
Sternum fracture	19	7.28

**Table 4:** Evaluation of the indoor and outdoor accidents according to the variables.

Parameter	Geriatric trauma		Statistical analysis
	Indoor (n = 136)	Outdoor (n = 125)	p-value
Age	75.93 ± 7.81	75.08 ± 7.77	0.377
Sex			0.229
Male	76 (55.9%)	79 (63.2%)	
Female	60 (44.1%)	46 (36.8%)	
Anticoagulant			0.02*
Yes	52 (38.2%)	31 (24.8%)	
No	84 (61.8%)	94 (75.2%)	
Trauma side			0.011*
Right	68 (50%)	50 (40%)	
Left	51 (37.5%)	41 (32.8%)	
Bilateral	17 (12.5%)	34 (27.2%)	
Number of rib fractures	4.66 ± 3.14	4.49 ± 2.53	0.628
Sternum fracture			0.063
Yes	6 (4.4%)	13 (10.4%)	
No	130 (95.6%)	112 (89.6%)	
Complication			0.178
Yes	67 (49.3%)	72 (57.6%)	
No	69 (50.7%)	53 (42.4%)	
Surgical intervention			0.211
Yes	31 (22.8%)	37 (29.6%)	
No	105 (77.2%)	88 (70.4%)	
Length of hospitalization (days)	4.38 ± 2.20	4.90 ± 2.89	0.106
Drainage time (days)	4.68 ± 1.76	4.12 ± 1.69	0.241
Extrathoracic trauma			0.010*
Yes	38 (27.9%)	54 (43.2%)	
No	98 (72.1%)	71 (56.8%)	
Intubation			0.623
Yes	3 (2.2%)	1 (0.8%)	
No	133 (97.8%)	124 (99.2%)	
Mortality			0.623
Yes	3 (2.2%)	1 (0.8%)	
No	133 (97.8%)	124 (99.2%)	
GCS score	14.77 ± 1.26	14.94 ± 0.3	0.145

## References

[1] Shi LL Pudney J Brangman S Parham K Nuara M Head & neck trauma in the geriatric population Otolaryngologic Clinics of North America 2023 56 6 1183 1201 10.1016/j.otc.2023.05.005 37385861

[2] Neupane I Wice M Monteiro JFG Lueckel S Kheirbek T Impact of geriatric trauma co-management on 1-year mortality in older adults with multiple rib fractures Rhode Island Medical Journal 2013 106 4 19 24 37098142

[3] Çubukçu M Falling risk assesment in home care patients Türk Aile Hekimliği Dergisi 2018 22 2 50 57 (in Turkish with an abstract in English). 10.15511/tahd.18.00250

[4] Kim SH Risk factors for severe injury following indoor and outdoor falls in geriatric patients Archives of Gerontology and Geriatrics 2016 62 75 82 10.1016/j.archger.2015.10.003 26553485

[5] Moncada LVV Mire LG Preventing falls in older persons American Family Physician 2017 96 4 240 247 28925664

[6] Kuş B Büyükyılmaz F Ardıç A Comparison of three fall risk assessment tools in older hospitalized patients in Turkey: Analysis of sensitivity and specificity Aging Clinical and Experimental Research 2023 35 5 1033 1041 10.1007/s40520-023-02369-z 36859749

[7] Barlow P A practical review of the Glasgow Coma Scale and Score The Surgeon 2012 10 2 114 119 10.1016/j.surge.2011.12.003 22300893

[8] Özdemir S Köse S Thoracic trauma and mortality in geriatric Turkish population: 6-month follow-up study General Thoracic and Cardiovascular Surgery 2021 69 3 504 510 10.1007/s11748-020-01507-y 33057969

[9] Pang I Okubo Y Sturnieks D Lord SR Brodie MA Detection of near falls using wearable devices: a systematic review Journal of Geriatric Physical Therapy 2019 42 1 48 56 10.1519/JPT.0000000000000181 29384813

[10] Gaddikeri MB Nene A Patel P Bamb H Bhaladhare S Sarcopenia and its effects on outcome of lumbar spine surgeries European Spine Journal 2024 33 4 1369 1380 10.1007/s00586-024-08155-3 38433166

[11] Stawicki SP Grossman MD Hoey BA Miller DL Reed JF 3rd Rib fractures in the elderly: a marker of injury severity Journal of The American Geriatrics Society 2004 52 5 805 808 10.1111/j.1532-5415.2004.52223.x 15086666

[12] Pyke OJ Rubano JA Jr Vosswinkel JA McCormack JE Huang EC Admission of elderly blunt thoracic trauma patients directly to the intensive care unit improves outcomes The Journal of Surgical Research 2017 219 334 340 10.1016/j.jss.2017.06.054 29078902

[13] Barry R Thompson E Outcomes after rib fractures in geriatric blunt trauma patients American Journal of Surgery 2018 215 6 1020 1023 10.1016/j.amjsurg.2018.03.011 29548528

[14] Hefny AF Abbas AK Abu-Zidan FM Geriatric fall-related injuries African Health Sciences 2016 16 2 554 559 10.4314/ahs.v16i2.24 27605971 PMC4994555

[15] Al-Aama T Falls in the elderly: spectrum and prevention Canadian Family Physician 2011 57 7 771 776 21753098 PMC3135440

[16] World Health Organization Ageing And Life Course Unit WHO global report on falls prevention in older age Geneva, Switzerland World Health Organization 2008

[17] Arslan Ö Tosun Z Comparison of the psychometric properties of three commonly used fall risk assessment tools: a prospective observational study for stroke patients Topics in Stroke Rehabilitation 2022 29 6 430 437 10.1080/10749357.2021.2008598 34850668

[18] Montero-Odasso MM Kamkar N Pieruccini-Faria F Osman A Sarquis-Adamson Y Evaluation of clinical practice guidelines on fall prevention and management for older adults: a systematic review The Journal of American Medical Association Network Open 2021 4 12 e2138911 10.1001/jamanetworkopen.2021.38911 PMC8674747 34910151

[19] Lord SR Close JCT New horizons in falls prevention Age and Ageing 2018 47 4 492 498 10.1093/ageing/afy059 29697780

